# The In Vitro Replication, Spread, and Oncolytic Potential of Finnish Circulating Strains of Herpes Simplex Virus Type 1

**DOI:** 10.3390/v14061290

**Published:** 2022-06-13

**Authors:** Kiira Kalke, Julius Orpana, Tuomas Lasanen, Olaya Esparta, Liisa M. Lund, Fanny Frejborg, Tytti Vuorinen, Henrik Paavilainen, Veijo Hukkanen

**Affiliations:** 1 Institute of Biomedicine, Faculty of Medicine, University of Turku, 20520 Turku, Finland; kiira.m.kalke@utu.fi (K.K.); jamorp@utu.fi (J.O.); tuaula@utu.fi (T.L.); olayaesparta@gmail.com (O.E.); liilun@utu.fi (L.M.L.); fanny.o.frejborg@utu.fi (F.F.); tytti.vuorinen@tyks.fi (T.V.); 2 Orion Pharma, R&D, Orion Corporation, 20380 Turku, Finland; 3 Drug Research Doctoral Programme (DRDP), University of Turku, 20520 Turku, Finland; 4 Clinical Microbiology, Turku University Hospital, 20520 Turku, Finland

**Keywords:** herpes simplex virus type 1 (HSV-1), oncolytic virus, oHSV, circulating strain, clinical isolate, phenotype, genotype, gI, gB

## Abstract

Herpes simplex virus type 1 (HSV-1) is the only FDA- and EMA- approved oncolytic virus, and accordingly, many potential oncolytic HSVs (oHSV) are in clinical development. The utilized oHSV parental strains are, however, mostly based on laboratory reference strains, which may possess a compromised cytolytic capacity in contrast to circulating strains of HSV-1. Here, we assess the phenotype of thirty-six circulating HSV-1 strains from Finland to uncover their potential as oHSV backbones. First, we determined their capacity for cell-to-cell versus extracellular spread, to find strains with replication profiles favorable for each application. Second, to unfold the differences, we studied the genetic diversity of two relevant viral glycoproteins (gB/UL27, gI/US7). Third, we examined the oncolytic potential of the strains in cells representing glioma, lymphoma, and colorectal adenocarcinoma. Our results suggest that the phenotype of a circulating isolate, including the oncolytic potential, is highly related to the host cell type. Nevertheless, we identified isolates with increased oncolytic potential in comparison with the reference viruses across many or all of the studied cancer cell types. Our research emphasizes the need for careful selection of the backbone virus in early vector design, and it highlights the potential of clinical isolates as backbones in oHSV development.

## 1. Introduction

Genetic variation exists between herpes simplex virus type 1 (HSV-1) clinical isolates, with differences ranging between 2–4% on a nucleotide level [1,2,3]. Based on their genotypes, HSV-1 isolates may be divided into three geographic clusters, the Eastern, Western, and Southern clades [2,3,4]. The clinical isolates vary from each other also by phenotype, such as by viral fitness and response to antivirals [3,5,6,7]. In most cases, the observed phenotype cannot be predicted by genotype, as viral phenotype has not been yet connected to any genomic groups nor any pattern of geographic diversity [3,8]. Searching for clinical isolates with phenotypes of interest requires relying on in vitro or in vivo screening for finding relevant viral attributes rather than on genotyping.

To date, HSV-1 represents the only approved modality for oncolytic virotherapy in the US and in Europe. The European Medicines Agency (EMA) and the Food and Drug Administration (FDA) have approved Imlygic, a replication competent oncolytic HSV (oHSV) used as a repetitive inoculation directly to the tumor to treat melanoma. Like the other oHSVs in the current clinical pipeline (Table 1), Imlygic is rendered safe by a deletion in the neurovirulence gene, γ_1_34.5, coding for ICP34.5 neurovirulence factor. The underlying mechanism is reversion of protein kinase R activity, which, in the absence of ICP34.5, blocks virus replication in noncancerous cells [9,10].

In addition to the aforementioned possibility of gaining clinical safety with the attenuation of one gene, HSV also boasts other features that separate this delivery modality from other approved viral platforms. In short, (1) the genome does not integrate, (2) it can harbor large transgenes, (3) it is neurotropic, (4) it has built-in small molecule drug sensitivity, (5) HSV is relatively easy to produce at an industrial scale as a replication competent vector, and (6) pre-existing immunity does not prevent repetitive local delivery [11]. Inspired by the unique features of oHSVs and the success of Imlygic, many other oHSVs have been entered into clinical trials for various treatment indications, such as melanoma, glioblastoma, or colorectal cancer (Table 1).

When designing an oHSV, many aspects must be taken into account, including the safety or efficacy resulting from the gene deletions, the possible transgene(s), the potential for combinatorial treatment, and additional delivery or targeting methods (reviewed in [23]). However, the aspect of choosing the parental virus is often neglected. A majority of the oHSVs in the current clinical pipeline originate from laboratory reference strains, such as HSV-1(F), KOS, or 17+ (Table 1). However, the serially passaged reference strains, which can represent certain enriched phenotypes or minor variants in cell culture [24], may harbor decreased oncolytic capacity in contrast to circulating strains of HSV-1, which have only limited passages in cultured cells.

The early design of the thus far only approved oHSV, T-Vec (Imlygic), began with the comparison of two clinical strains for their oncolytic potential [5]. Both of the clinical strains were found to be more oncolytic than 17+, and the more oncolytic clinical strain, JS-1, was chosen as the parental strain for oHSV development. The increased oncolytic potential was retained even after the chosen clinical isolate, JS-1, was compared after its neurovirulence gene deletion [5]. Despite the success of the JS-1 based Imlygic, only a few other companies have utilized circulating strains as their oHSV parental strains. However, as circulating strains differ in geno- and phenotype [3], the optimal process would involve the screening of many clinical isolates to find those with more potential than the already well-characterized reference strains. An example, for RP1, RP2, and RP3 oHSVs (Table 1), 29 clinical isolates were compared in vitro before choosing the RH018 strain as the parental strain for oHSV development [6].

In this study, we compare 36 clinical HSV-1 isolates from Finland with respect to their potential as parental strains for future oHSV or HSV gene delivery vector development. Based on our results, we can conclude that the spread and the replication profiles as well as the oncolytic potential of the circulating isolates are highly dependent on the cell type. However, we were able to identify circulating strains with increased oncolytic properties in contrast to all studied reference viruses (17+, F, KOS) across many or all of the studied cancerous cell types. This observation highlights the potential of clinical isolates as oHSV backbones as opposed to reference strains, and it emphasizes the need for careful selection of the backbone virus in early vector design, depending on the future implementation of the HSV construct.

## 2. Materials and Methods

### 2.1. Cell Lines

Five different cell lines were used in this experiment. U373MG cells, currently reclassified as U251 (HTB-17, ATCC, Manassas, VA, USA) but here referred to with their original name for continuity with earlier publications from the group, were maintained in DMEM (Lonza, Basel, Switzerland) with 5% heat inactivated fetal bovine serum (FBS), 1% GlutaMAX (Gibco, Carlsbad, CA, USA), and gentamycin. HCE cells (kindly provided by Arto Urtti from the University of Helsinki and the University of Eastern Finland, Kuopio, Finland) and SW480 cells (CCL-228, ATCC, Manassas, VA, USA) were maintained in the same medium as the U373MG cell line. Vero cells (CCL-81, ATCC, Manassas, VA, USA) were maintained in M199 medium (Gibco), 5% heat inactivated FBS, and gentamycin. Raji cells (CCL-86, ATCC, Manassas, VA, USA) were maintained in RPMI 1640 (Lonza) with 10% heat inactivated FBS, 1% GlutaMAX, and gentamycin.

### 2.2. Viruses

In this study, we used 36 anonymous archival HSV isolates from the Department of Virology, University of Turku. The clinical isolates were of early passage (passage 2–4) and had previously been confirmed as HSV-1 with an immunoperoxidase rapid culture assay [25]. Additionally, HSV-1 reference strains 17+, F, KOS, an ACV-resistant, thymidine kinase deficient strain (HSV-1 Δ305) [26], and neurovirulence gene deleted H1052 [27] representing an oHSV backbone, were used as controls in the experiment. The virus stocks used in the experiments of this research article were propagated in Vero cells by culturing the viruses in T25 flasks until full cytopathic effect, freezing the cells and supernatant in milk, and lysing the cells by a twice-repeated freeze-and thaw cycle (−80 °C, +37 °C) before the determination of the concentration of the virus stock. For a summary of viruses used, please see Table 2.

### 2.3. Acyclovir Sensitivity Assay

ACV sensitivity was studied with a plaque reduction assay on Vero cells, as before [3,7]. The assay was conducted in a 96-well plate format with infections of 50 pfu/well and an acyclovir concentration ranging from 128 µg/mL to 0.03 µg/mL. At 2 h post infection (hpi), medium supplemented with human IgG (80 mg/L) was added to the cells, and at 72 hpi, the cells were fixed with methanol, stained with 0.1% crystal violet, and the plaque counts were determined. The limit of acyclovir resistance was considered to be the half maximal inhibitory concentration (IC_50_) value of 1.9 μg/mL.

### 2.4. Virus Yield

To determine the viral yield of each viral strain, Vero cells were infected with 5 plaque forming units (pfu) per cell of each virus on a 96-well plate. Samples were taken from the culture supernatant at 6, 24, 48, and 72 hpi. A plaque assay was used to determine the titers at each time point.

### 2.5. Assay Comparing Cell-Associated and Extracellular Virus

For each cell line, the concentrations of both the extracellular and the cell-associated virus were determined in Vero, U373MG, and HCE cells, as previously described [3]. The samples were taken at 24 hpi by collecting the supernatant on a separate 96-well plate and covering the cells with 9% autoclaved milk in PBS or in 10% FBS. Before analysis of cell-associated virus titers, the cells were lysed by a thrice-repeated freeze-and thaw cycle (−80 °C, +37 °C). The samples were quantified for virus titer by plaque assay.

### 2.6. Sequencing

Viral genes UL27 and US7, encoding the glycoproteins B (gB) and I (gI), respectively, were sequenced for areas with maximal variability between 17+ (JN555585.1) and the recently full-genome sequenced Finnish clinical isolates [3]. The primers designed for UL27, 5′-CGGTGGTCTCCAGGTTGTTG-3′ (reverse) and 5′-TGGTCTACGACCGAGACGTT-3′ (forward), were used to sequence nucleotides 55,132–155,897 according to the numbering of the 17+ strain sequence, JN555585.1. The primers designed for US7, 5′-ACGTGTTACGCGTATGGGTC-3′ (forward) and 5′-TATACCAACAGGGGAGGCGT-3′ (reverse), were used to sequence nucleotides 140,222–140,960 according to the numbering of the 17+ strain sequence JN555585.1. The target sequences were amplified from viral stocks using Phusion polymerase (Thermo Scientific, Waltham, MA, USA). The product from the PCR was confirmed with an analytical agarose gel run. The resulting DNA from the PCR run was purified using a GeneJET PCR Purification Kit (ThermoFisher, Waltham, MA, USA), according to the manufacturer’s protocol. The purified DNA was sequenced by LightRun, Eurofins Genomics, Ebersberg, Bayern, Germany.

### 2.7. Cytolytic Assay for Determination of Oncolytic Potential

The oncolytic potential of the strains was determined with a test for cytotoxicity, CellTiter-Glo^®^ viability assay (Promega, Madison, WI, USA). The assay was conducted on a 96-well plate format for U373MG, SW480, and Raji cells with 2 pfu/cell infections. At 96 hpi, the cellular viability was quantified with the CellTiter-Glo^®^ viability assay according to the manufacturer’s instructions. The resulting cellular viability representing the oncolytic effect for each strain was determined against untreated samples.

### 2.8. Statistical Analysis

Statistical analyses were performed with SPSS statistics v.25.0.0.1 (IBM, Armonk, NY, USA). The statistical significances were calculated with Mann–Whitney’s non-parametric U-test by comparing two individual groups at a time, with the threshold of significance set as a *p*-value of < 0.05. The sigmoidal dose–response curves and their associated EC50 values were fitted and calculated with Origin 2016 v.b9.2.3.303 (Academic) (OriginLab Corporation, Northampton, MA, USA). The sequences were aligned with ClustalW-algorithm and the phylogenetic trees were created in ML-tree format, utilizing the Bootstrap method with 1000 replicates.

## 3. Results

### 3.1. Comparison of Growth Properties of the Circulating Strains In Vitro

**Figure 1:**
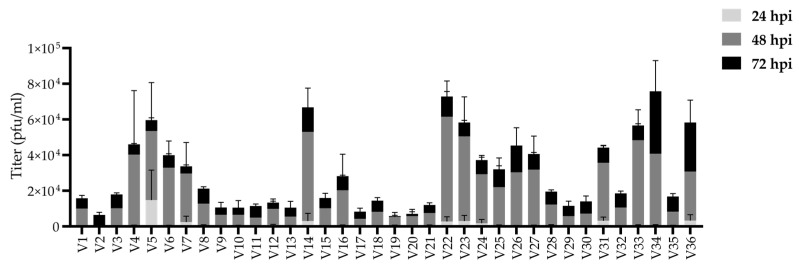
Cumulative virus yield of the clinical isolates in Vero cells. Vero cells were infected at 5 pfu/cell and quantified for any extracellular virus shed to the supernatant at 24, 48, and 72 h post infection (hpi). The yield of the virus in the supernatant is presented as stacked bars, so that the shed virus amounts from each time point are stacked on top of the yield from the previous time point. The light gray section (bottom section) represents mean of the virus yield at 24 hpi, the dark gray section (middle section) represents mean of the virus yield at 48 hpi, and the black (top section) section represents the mean of virus yield at 72 hpi. The whiskers represent the standard deviation of the mean for each time point (*N* ≥ 4 per clinical isolate). The data is derived from four or more biological replicates for each clinical isolate.

To determine the virus yield from the clinical isolates in Vero cells, the cells were infected with 5 pfu/cell and a supernatant sample was collected at 24, 48, and 72 h post infection (hpi) to quantify the released virus. The results are presented in a stacked graph (Figure 1). All of the clinical isolates replicated in Vero cells. The isolates V2, V19, and V20 represented the isolates with the three lowest, and V14, V22, and V34 represented the three highest overall yields. The profiles of the yields were similar between a majority of the isolates, with the most abundant shedding at the 48 h time point and plateauing at the 72 h time point. Hence, the majority of the viral production took place between the 24- and 48-h time points. The evident outliers of the group were isolate V2, which displayed abundant shedding later than other isolates, peaking at 72 hpi, and isolate V5 for which the yield at 24 hpi was higher than of any other isolate.

**Figure 2:**
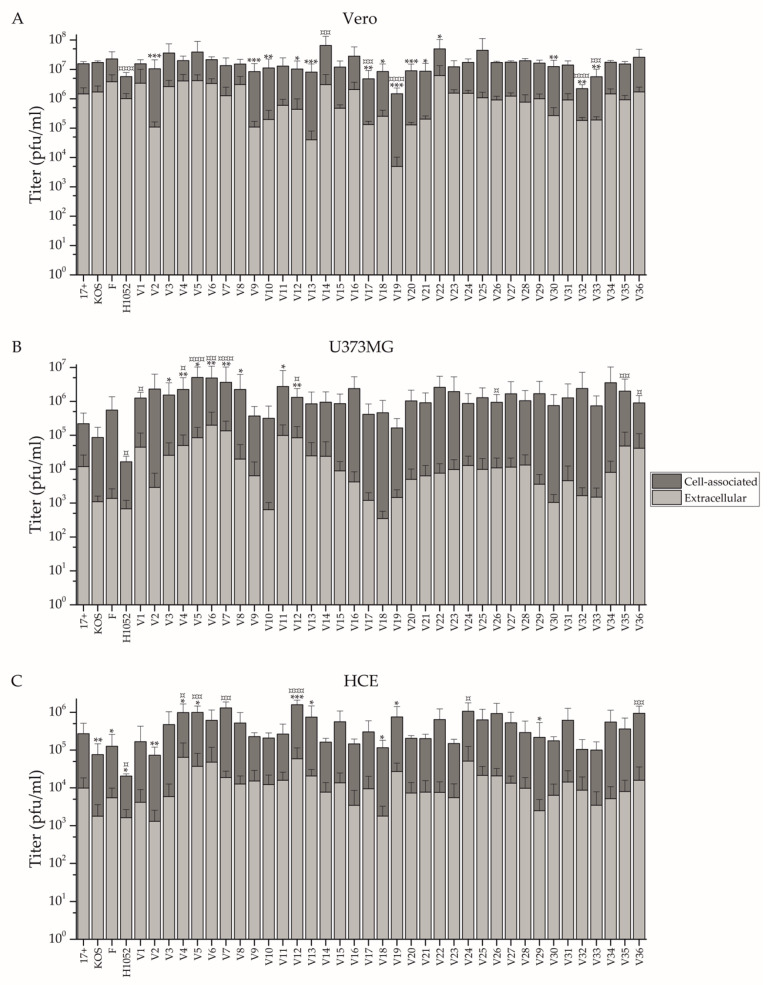
The replication profiles of the clinical isolates, based on extracellularly released progeny and cell-associated virus. The light gray bars depict the extracellular titer and the dark gray bars the cell-associated titer. The bars are shown overlaid on each other to emphasize how cell-associated titers are consistently higher than extracellular titers. (**A**) Vero, (**B**) U373MG, and (**C**) HCE cells were infected with 0.1 pfu/cell of each clinical isolate. At 24 h post infection (hpi), samples of extracellular virus shed to the supernatant, and cell-associated virus bound in the cells were taken separately from each sample. The extracellular and cell-associated titers were then determined with a plaque assay. The bars represent the titers in pfu/mL. The whiskers represent the standard deviation of the mean (*N* ≥ 8 per clinical isolate, data from two individual experiments). The reference virus 17+ was used for pairwise comparisons separately for extracellular (*, *p* < 0.05; **, *p* < 0.01; ***, *p* < 0.001) and cell-associated virus (¤, *p* < 0.05; ¤¤, *p* < 0.01; ¤¤¤, *p* < 0.001) titers.

The spread of the clinical isolates was studied in human corneal epithelial (HCE), astrocytoma (U373MG) and Vero cells by determination of cell-associated and extracellular titers separately at 24 h after a 0.1 pfu/cell infection. The titers are presented in overlaid columns in Figure 2, and the ratios of the cell-associated and released virus are in Table 3. In all studied cell types, the cell-associated titers, reflecting the amount of the cell-bound virus, were constantly higher than the extracellular titers (Figure 2).

In Vero cells (Figure 2A) strains V2, V9, V10, V12, V13, V17, V18, V19, V20, V21, V30, V32, and V33 had significantly lower extracellular titer than the reference strain 17+, while only strain V22 had a higher titer (Figure 2). Cell-associated titers were more equal in Vero cells, as only strains V17, V19, V32, V33, and the recombinant H1052 had a significantly lower titer than 17+, while V14 had a significantly higher titer than 17+ (Figure 2). The extracellular virus titer was equivalent to 0.6% (V13) to 90.5% (V8) of the cell-associated virus titer, with no strain being significantly more prone to release virus than 17+, but with V2, V9, V12, V13, V19, V20, V21, V26, and V30 all being significantly more cell-associated strains than 17+ (Table 3).

In U373MG cells (Figure 2B), no isolates had a significantly lower extracellular titer than 17+, however, strains V3, V4, V5, V6, V7, V8, V11, and V12 had a significantly higher titer. Isolates V1, V4, V5, V6, V7, V12, V26, V35, and V36 had significantly higher cell-associated titers. Only the recombinant H1052 possessed a significantly lower titer than 17+. The extracellular virus titer was equivalent to 0.1% (V18, V29, V33) to 7.4% (V7) of the cell-associated virus titer, with no strain having a significantly more cell-associated phenotype than 17+, but with V7 and V12 being significantly more of a shedding phenotype than 17+ (Table 3).

In HCE cells (Figure 2C), strains V2, V18, V29, H1052, KOS, and F had a significantly lower extracellular titer than 17+, while V4, V5, V12, V13, and V19 had a higher titer. Only H1052 had a significantly lower cell-bound titer than 17+, while V4, V5, V7, V12, V24, and V36 had a higher cell-bound titer. The extracellular virus titer was equivalent to 1.3% (V29) to 13.3% (V4) of the cell-associated virus titer, with no strain having a significantly more shedding phenotype, but with V29 and V36 being significantly more cell-associated than 17+ (Table 3).

Isolates V4, V5, and V12 all yielded a significantly higher titer for released and cell-associated virus in both HCE and U373MG cells, whereas V7 and V36 yielded significantly higher titers for cell-associated virus amount in both HCE and U373MG cells (Figure 2). The only strain to have a significant difference in released to cell-associated virus proportion in more than one cell line is V12, which was more cell-associated than 17+ in Vero cells and less cell-associated than 17+ in U373MG cells. Standing out from Table 3 as having the lower proportions of released to cell-bound virus in all three cell lines is V2, whereas V1 and V4 stand out as having the highest proportions in all three cell lines (Table 3).

The reference viruses KOS and F were rather similar to the reference virus 17+ used as a point of comparison, with the exception that in HCE cells they yielded lower titers of released virus than 17+ (Figure 2). The recombinant, 17+ based neurovirulence gene deleted H1052 was different from 17+ in all studied cell lines, having a significantly lower cell-bound titer across the cell lines and a significantly lower titer for released virus in HCE cells. Furthermore, H1052 stands out as one of the highest relative shedders across all three cell lines (Table 3).

**Figure 3:**
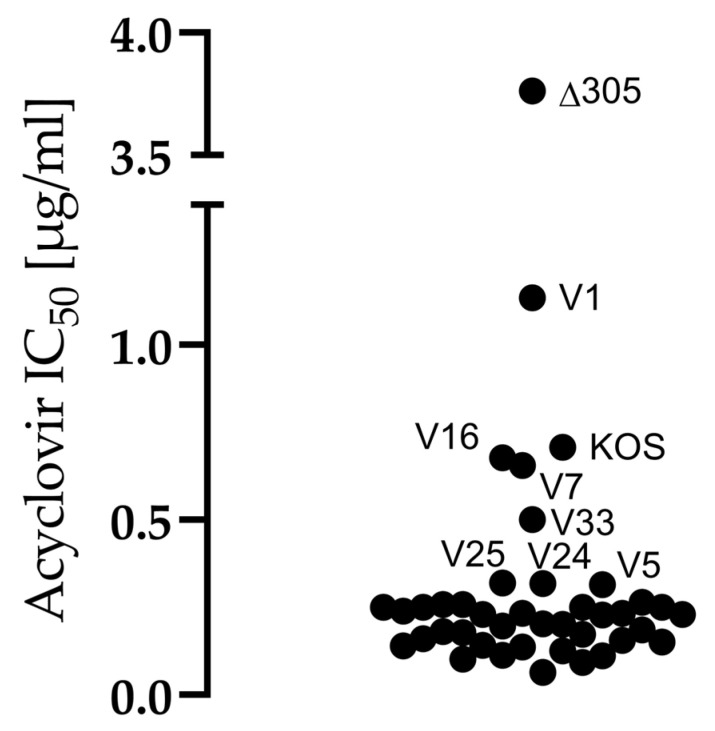
Sensitivities of the strains to acyclovir. Sensitivity to acyclovir (ACV) was measured with an assay conducted in Vero cells. For the assay, the cells were pre-treated with ACV (0.03125–0.03128 µg/mL) and subsequently infected with 50 pfu of each virus in duplicates in a 96-well format. The read-out was at 72 h post infection (hpi), when the formed virus plaques were quantified. Based on the amount of plaques in the control wells, half-maximal inhibitory values (IC_50_) for ACV were determined separately for each strain. The limit of resistance was considered to be an IC_50_ value of >1.90 µg/mL. The IC_50_ value of each strain is plotted in the figure, and the names of the strains separating from the group are shown. The strain Δ305, which is a thymidine kinase deficient strain, was included as an ACV resistant control.

To study the susceptibility of the clinical isolates, reference strains and H1052 to acyclovir, an acyclovir sensitivity assay was conducted. The limit of acyclovir sensitivity was considered to be the half-maximal inhibitory concentration (IC_50_) of 1.90 µg/mL, as before [3]. The acyclovir-resistant, thymidine kinase deficient strain Δ305 exceeded the level of resistance with an IC_50_ value of 3.7 µg/mL. As the IC_50_ values for the clinical strains ranged from 0.14 µg/mL to 1.13 µg/mL, none of the isolates were resistant to acyclovir (Figure 3). Furthermore, the reference viruses and H1052 were sensitive to acyclovir. With the exception of Δ305, V1 was the least sensitive to acyclovir with an IC_50_ value of 1.13 µg/mL, followed by KOS, V7, and V16 with IC_50_ values of 0.76 µg/mL, 0.65 µg/mL and 0.65 µg/mL, respectively.

### 3.2. US7 and UL27 Genotypes of the Clinical Isolates

**Figure 4:**
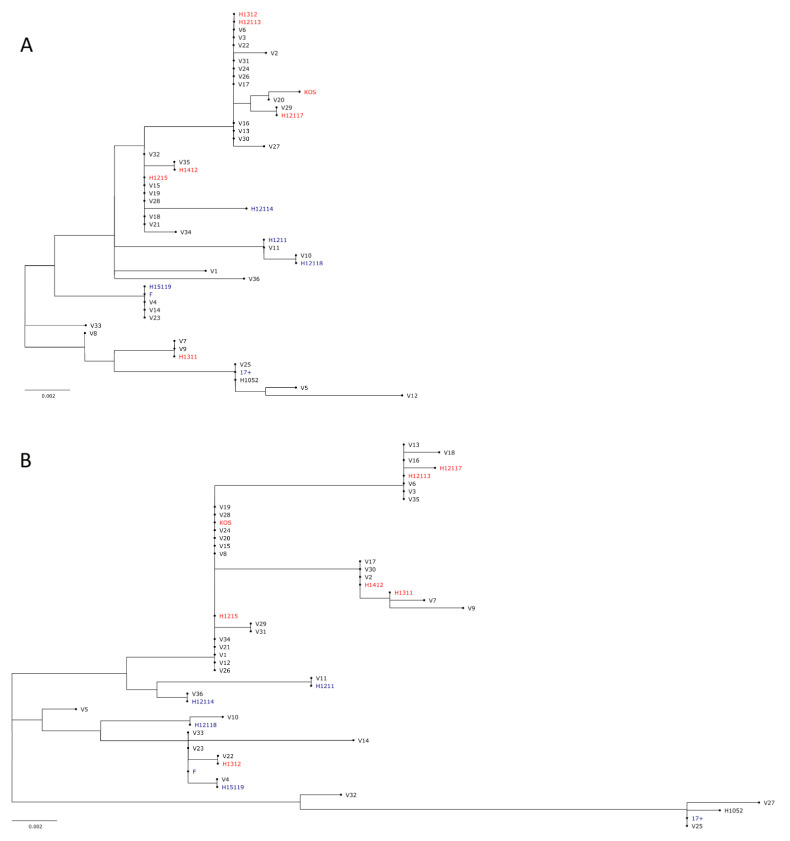
Genotypic relatedness of the studied strains by UL27 and US7 genes. Phylogenetic trees were constructed based on partial sequencing of the (**A**) UL27 gene and the (**B**) US7 gene, coding for gB and gI, respectively. All clinical isolates, F, KOS, 17+, and H1052 were sequenced. Additionally, previously full genome sequenced Finnish strains [3] were included in the analysis. For UL27, nucleotides 55,132–55,897 and for US7, nucleotides 140,222–140,960, were sequenced according to the numbering of Genbank submission JN555585.1 (HSV-1 strain 17+). The sequences were aligned with ClustalW-algorithm and the phylogenetic trees were created in ML-tree format utilizing the Bootstrap method with 1000 replicates. Strains with previously assigned Asian or European/North American clades [3] are colored with red or blue, respectively.

Two viral genes that contribute to cell-to-cell spread, UL27 and US7, were partially sequenced to find any genomic connections to the observed phenotypes. The sequenced part was chosen based on the maximal diversity of the gene sequence in the previously full genome sequenced Finnish clinical isolates [3]. There was evident diversity in the studied sequences of the clinical isolates. All of these found genomic differences allowed for studying the genomic relatedness of the strains, as displayed in Figure 4 with phylogenetic trees.

The differences in US7 were more abundant than those of UL27 (Figure 4, Supplementary Files S1 and S2). In the sequenced region of UL27 the isolates had both unique SNPs when compared to the consensus of all clinical isolates as well as SNPs common for a group of clinical isolates (Supplementary File S1). In the sequenced region of US7, there were also both unique SNPs as well as SNPs repeating in many clinical isolates (Supplementary File S2). Furthermore, in US7, there were a varying number of an intragenic repeat regions (CCTCCACCCCCTCGACCACCA, [4]) in the clinical isolates: five in V2, four in V5, V10, V22, V23, V30, and V33, three in V1, V4, V7, V11, V12, V14, V17, V21, V32, V34, V36 and two in the rest (Supplementary File S2). There were minimal SNPs in the repeats, but the first repeat of V9 had one SNP (CCTCCACCCCCTCAACCACCA, SNP underlined), and, similar to H1211 [3], the first repeat of V11 had two (CCTTCACCCCCTCAACCACCA, SNPs underlined). The reference strains KOS, F, and 17+ had 2, 3, or 2 repeats, respectively. In 17+, a sequence following the repeat (TCCCCGCTCCCTCGACCACCA), which was detected before in 17+ [4], was observed again in 17+, but also in V25 and H1052 (Supplementary File S2).

There is no obvious grouping in either the US7 or the UL27 genotype in the sequenced regions, but there is some evident grouping with the reference strains in both genes (Figure 4). Altogether, the clinical isolates do show relatedness to the reference strains as well as to the previously published Finnish genotypes (published in [3]), for example, KOS groups with V20, 17+ groups with V25, F groups with V23, H12118 groups with V10, H1211 groups with V11, H1311 groups with V7 and V9, H1215 groups with V34 and V21, and H12113 groups with V3 and V6 according to both studied genes. However, there are also strains at distal locations to each other according to one and proximal to each other according to the other sequenced gene, such as H12113 and H1312, H1412 and V35, and V12 and V26.

To assess whether the strains can be divided by geographical genotypes, the Finnish strains with the known geographic associations were included in the analysis (Asian or European/North American, colored with red or blue, respectively, in Figure 4). Based on the grouping of these full-genome sequenced strains (published in [3]), there could be a tendency of the strains to group to the Asian or European/North American clades by the partial sequencing of UL27 and US7. In UL27 (Figure 4A), the grouping to Asian vs. European/North American clades is disrupted by H1311 and H12114, which group closer to the opposite clade. In US7 (Figure 4B), the only strain disrupting the hypothesis is H1312. This hypothesis would mean that 12 of the clinical isolates were of the European/North American clade and 24 would be of the Asian clade.

### 3.3. Oncolytic Potential

**Figure 5:**
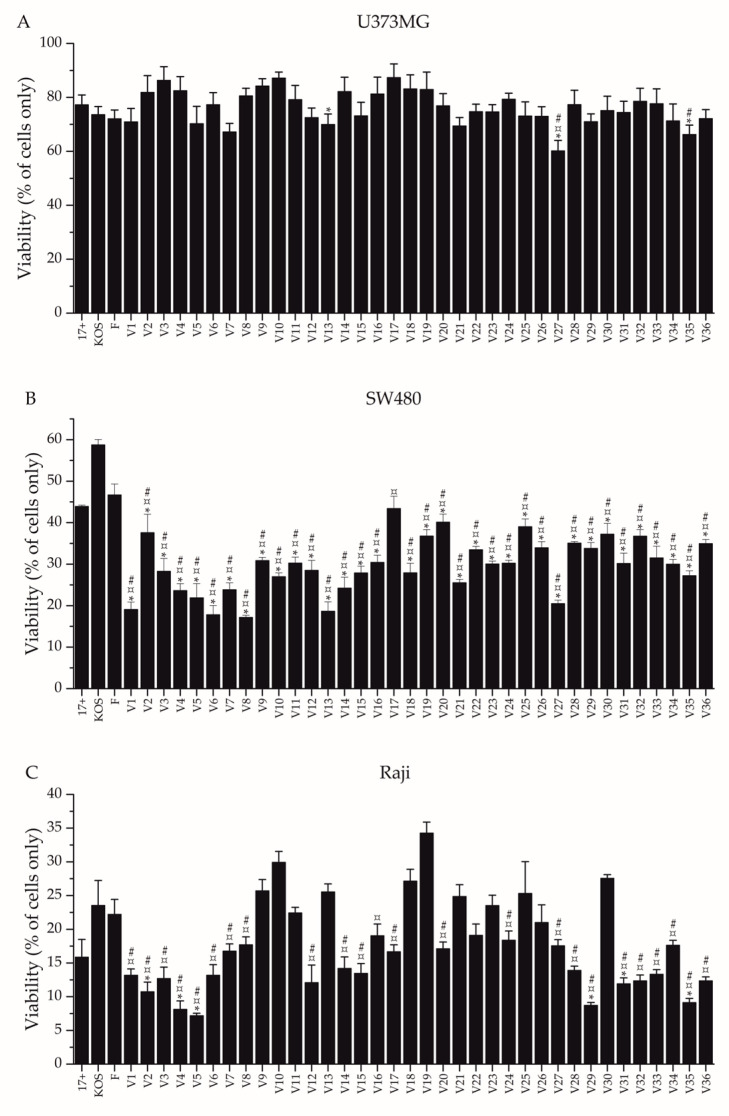
Cytotoxicity in selected cancer cell lines. The oncolytic effect of each clinical isolate and reference strain was determined in a cancerous cell line representing (**A**) neuroglioma (U373MG), (**B**) adenocarcinoma (SW480), and (**C**) lymphoma (Raji). The cells were infected with 2 pfu/cell of each strain and measured for viability at 4 days post infection, utilizing a luminescent assay. The viability was determined by comparing the signal derived from the infected cells to that derived from uninfected cells. The bars represent the percentile viability of the cells, while the whiskers represent the standard deviation of the mean (*N* ≥ 4 per each condition). Statistical differences against each of the reference strains were determined by pairwise comparisons. Any statistical differences found in pairwise comparisons with the reference strains are presented: against 17+ with an asterisk (*, *p* < 0.05), against F with a number sign (#, *p* < 0.05), and against KOS with a currency sign (¤, *p* < 0.05).

To study the oncolytic potential of the strains in cancerous cell types, we used a luminescent assay to determine cytotoxity of each of the strains to cells representing neuroglioma (U373MG), adenocarcinoma (SW480), and B-cell lymphoma (Raji) (Figure 5). After a 2 pfu/cell inoculation and 4 days of incubation, all strains had decreased the viability of each of the cancer cell types (Figure 5).

The cytolytic effect of the clinical isolates was most pronounced in Raji and SW480 cells, where cellular viability decreased approximately by 65–95% and by 55–85%, respectively. For U373MG cells, the viability decrease was 10–40%. In U373MG and Raji cells, the reference strains were within the range of the clinical isolates. However, in SW480 cells, all reference strains had a weaker effect on viability than those by any of the clinical isolates (Figure 5). There were no differences between the cytotoxicities of the reference strains in U373MG cells. In SW480 cells, KOS was significantly less oncolytic than F and 17+, but there was no statistical difference between 17+ and F. In Raji cells, both KOS and F were significantly less oncolytic than 17+, but there was no statistical difference between KOS and F.

In U373MG cells (Figure 5A), three clinical isolates (V13, V27, V35) were more oncolytic than 17+, one isolate (V27) was more oncolytic than KOS, and two isolates (V27, V35) were more oncolytic than F. In U373MG cells, there were also many isolates which were less oncolytic than the reference viruses, with V10, V14, and V17 being less cytotoxic than all three reference viruses (Figure 5). In SW480 cells (Figure 5B), all isolates but V17 were more oncolytic than 17+ and F, whereas all isolates, with no exception, were more oncolytic than KOS. In Raji cells (Figure 5B), six clinical isolates (V2, V4, V5, V29, V31, V35) were more oncolytic than 17+, twenty-four isolates (V1-V8, V12, V14-V17, V20, V24, V27-V29, V31-V36) were more oncolytic than KOS all of which but V16 were also more oncolytic than F. H1052 was equally oncolytic to 17+ in U373MG cells, less oncolytic to 17+ in SW480 cells and more oncolytic to 17+ in Raji cells (Figure S1).

## 4. Discussion

### 4.1. Replication and Spread of the Clinical Isolates Is Dependent on the Cell Type

The yield of extracellular virus was studied in Vero cells (Figure 1), the most common cell line for HSV propagation. The spreading capability of the strains by cell-associated or extracellular routes was studied in a human corneal epithelial (HCE) cell line and in a neuroglioma cell line (U373MG), representing natural target tissues of HSV, as well as in Vero cells (Figure 2, Table 3). The significant differences between the phenotypes of the clinical strains and the reference strains are summarized in Table 4.

All clinical isolates (V1-V36) replicated in Vero cells with relatively identical profiles, displaying the highest extracellular shedding at 2 days post infection and plateauing at 3 days post infection (Figure 1). In contrast to the majority of the clinical isolates, V2 was slower in phenotype, showing the highest absolute shedding later at the 3-day time point, whereas V5 was most rapid, reaching higher absolute yields than the other isolates at the 1 day post infection time point (Figure 1). This result is supported by the high titers of V5 also in other cell types (Figure 2, Table 4) and the highly cell-associated phenotype of V2, which diminishes the accumulation of virus in the culture supernatant (Figure 2, Table 3).

In all cell types and with all studied strains, the cell-associated virus titers were higher than the extracellular virus titers (Figure 2), demonstrating the cell-associated phenotype typical for wild type HSV-1. Among the studied cell types, the highest shedding and absolute titers were reached in Vero cells (Figure 2, Table 3), with few isolates displaying high relative viral shedding of over 50% (Table 3). The fact that Vero cells supported the most shedding among the cell types was no surprise, as Vero cells lack type I interferon response, and they are recognized as a cell line for HSV cultivation and manufacturing due to abundant viral shedding and rapid replication. In Vero cells, only one isolate released a significantly higher amount of extracellular virus than 17+, and no strain was significantly more prone to shed as 17+, as per the ratios to extracellular and cell-associated virus (Figure 2, Table 3). Likewise, out of all the clinical isolates, only one reached a significantly higher cell-associated titer than 17+ in Vero cells (Table 3). Nevertheless, by extracellular to shed virus ratio a fourth of the clinical isolates (9/36) were of a significantly more cell-associated phenotype in Vero cells than 17+ (Table 4). As the other reference strains, KOS and F, were similar to 17+, it is feasible to assume that the long in vitro evolution of these strains with Vero cells during passaging has favored for a more extracellular phenotype with high yields in absolute extracellular and cell-associated titers.

In the neuroglioma cell line (U373MG), KOS and F were similar to 17+ (Figure 2B, Table 3), but there were a total of five isolates that reached both a higher extracellular titer and a higher cell-associated titer than 17+ (Table 4). Of the five isolates, two (V7, V12) were also significantly more of the shedding phenotype than 17+ (Table 4), demonstrating an abundant replication of these two strains in U373MG cells. However, these two strains were not closely related by their gI nor gB genotype (Figure 4), nor was the efficient replication of any of the five isolates reflected to a significantly more cytotoxic phenotype in the cell line (Figure 5A).

In HCE cells, the reference viruses KOS and F were different from 17+, as they both yielded significantly lower titers of released virus (Figure 2C). However, the cell-bound titers (Figure 2C) and ratios of extracellular and shed virus (Table 3) were similar between the reference viruses. There were five clinical isolates with a higher extracellular and six isolates with a higher cell-bound titer in comparison to 17+, with V4, V5, and V12 being part of both groups (Table 4). Yet, though having both elevated extracellular and cell-associated titers, none of these four strains was different in their relative cell-bound or extracellular spreading in contrast to 17+ (Table 3). However, V36, which had an elevated cell-bound titer (Figure 2C), also had a significantly more cell-associated phenotype than 17+ (Table 3). The clinical isolates with the more cell-associated phenotypes or elevated extracellular or cell-associated titers were not similarly related by their gI or gB genotype (Figure 4). We consider that an isolate, such as V36, spreading efficiently and mainly from cell-to-cell in human corneal epithelial cells could serve as a potential parental strain for either gene therapy of the eye or in the treatment of corneal or conjunctival tumors. After selection of treatment indication, further assaying in vitro, preferably in human primary cells or other relevant models representing the target tissue, would be necessary to select between the candidates deemed most promising based on this study.

We expected that the clinical isolates, being more adapted to human epithelium, would have altogether demonstrated a more abundant infection in HCE cells. Therefore, it was surprising that so few clinical isolates displayed a more abundant replication in HCE cells in contrast to the reference strains. However, HCE cells represent the epithelium of the cornea and not the oral epithelium from where a majority of the strains are likely collected. Furthermore, the reference strains have been accustomed to the epithelial cell type, such as Vero cells, for a long time in vitro. Nevertheless, in Vero cells, hardly any clinical isolates were more efficient in replication than the reference strain 17+, whereas many strains with an elevated capability for replication and spread (Table 4) were identified in both cells of the nervous system (U373MG) and in cells of the human corneal epithelium (HCE). The result demonstrates the advantages which clinical isolates may harbor over the serially-passaged reference strains for therapeutic use in human cells.

Altogether, the replication characteristics of the isolates were dependent on the cell type (Figure 2, Table 3), as also concluded in previous research [3]. However, there were isolates which repeated their phenotype between cell types. As an example of this, V2 had a tendency for a more cell-associated phenotype than the other strains in all three cell lines (Table 3). Furthermore, V4, V5, and V12 all had elevated cell-associated and extracellular titers in both U373MG and HCE cells, demonstrating their high overall capability for a rapid and effective spread in these two cell types (Table 4). V7 was similar to V4, V5, and V12 in this sense, but it lacked elevated extracellular spread in HCE cells (Table 4). On the other end, V36 had elevated cell-associated titers in the same two cell types, without any significant elevation of extracellular virus (Table 4). The cell-associated spread is important, as an enhanced tendency for cell-to-cell spread would be preferred for oHSVs to allow efficient and lytic spread in solid tumors independent of tumor surface heterogeneity and with minimal contact to any neutralizing antibodies. These results could suggest that elevated cell-associated spread across cell lines is rather rare, but isolates with an overall elevated capability for replication are more probable to find. However, the choices of cell types, and of isolates, were limited.

### 4.2. All Clinical Isolates Were Sensitive to Acyclovir

All clinical isolates were sensitive to acyclovir (Figure 3). The acyclovir sensitivities of the isolates were well in line with previous research [3,7,32,33]. Though sensitive to acyclovir, isolate V1 stood out with a lower sensitivity than the other isolates (Figure 3). It is possible that under acyclovir selection with low concentrations, V1 may reveal a subpopulation resistant to acyclovir, which would be contraindicated to oHSV development.

### 4.3. The Phenotype Did Not Predict the US7 nor UL27 Genotype

Two genes contributing to cell-to-cell spread, UL27 and US7, encoding for gB and gI, respectively, were partially sequenced. The aim of the sequencing was to potentially explain some differences uncovered in the phenotypes of the clinical isolates (Figure 1, Figure 2, Figure 3, Figure 4 and Figure 5). The clinical isolates were different to each other in both of the studied genes, with more differences detected in the US7 sequence than in the UL27 sequence (Figure 4). Rather than grouping with each other only, the clinical isolates were grouping or pairing to proximity of previously full genome sequenced Finnish isolates [3] or reference strains, which were included in the phylogenic trees (Figure 4). This demonstrates that the studied clinical isolates do comprehensively represent the clinical isolates in Finland, and they are likely from varying geographical backgrounds. Therefore, we decided to check whether the domains we sequenced would reflect the determined European/North American and Asian clades of the previously full-genome sequenced Finnish isolates. Indeed, the phylogenetic trees reflected the European/North American and Asian division, with only one strain disrupting the hypothesis in both genetic areas (Figure 4). Assuming that the division to the geographical groups is as concluded, it would mean that 12 of the clinical isolates were of the European/North American clade, and 24 would be of Asian clade, which is a similar ratio for Finnish circulating strains as in previous literature [3]. Altogether, no evident groups of isolates repeating in both of the sequenced genes were formed, and the grouping of clinical isolates in the phylogenetic trees of US7 and UL27 were only partly similar (Figure 4), reflecting the moderate evolutionary relatedness in the two genes. In both sequenced areas, there were many SNPs, with some being shared between many isolates, and some being unique to the isolates (Figure 4, alignments in Supplementary Files S1 and S2). In addition to the SNPs, there was a varying number (*N* = 2–5) of a previously described US7 tandem repeat in the studied clinical isolates [4], the only strain with five repeats being V2, which displays a slow, highly cell-bound phenotype in contrast to 17+ (Figure 1 and Figure 2, Table 3). The higher amount of the tandem repeats could be a partial explanation behind the stand-out phenotype of V2, however, previously, the amount of tandem repeats has been thought not to have any connection to the phenotype [4] nor has the US7 genotype affected the phenotype [34]. Therefore, any confirmation of the connection between the repeats and the phenotype of V2 would require extensive further research, including full-genome sequencing of the isolate(s). No other relations between the uncovered phenotypes and the genotypes nor phylogenetic groups could be made. Therefore, as in previous research [3,8], the genotype did not predict phenotype.

### 4.4. The Clinical Isolates Could Be Advantageous as oHSV Backbones

The oncolytic potential of the strains was studied in cancerous cell types representing neuroglioma (U373MG), adenocarcinoma (SW480), and B-cell lymphoma (Raji) (Figure 5). After a 2 pfu/cell infection and 4 days of incubation, all isolates had decreased the viability of each of the cancer cell types, with most pronounced effects detected in Raji cells and least pronounced effects detected in U373MG cells (Figure 5). In our panel of cancer cell lines, the oncolytic potential of the isolates and how they performed in comparison to the reference strains or to each other was dependent on the cell type (Figure 5). However, in previous research [5], the relative differences in the oncolytic potential of the compared two clinical strains and 17+ actually remained across cell lines representing breast adenocarcinoma, colorectal adenocarcinoma, and melanoma, although no statistical significances of improved oncolytic capability were reported. In another previous research article, eight isolates, scored most lytic of twenty-nine clinical isolates, were compared in seven cancer cell types by visual assessment for their lytic capabilities [6]. Of the eight, one isolate was evidently most lytic across the cell types, but the oncolytic capability of others appeared dependent on the cell type. Even though the differences were assessed by visual ranking, which might be subjective and thus suboptimal, these results were similar to ours, as we also found the oncolytic capability of the strains to be rather dependent on the cell type but identified some outstanding oncolytic candidates. Altogether, in our study, numerous clinical isolates that were significantly more oncolytic than some or all of the reference strains could be identified in each cancer cell type (Figure 5). Notably, there were two clinical isolates, V27 and V35, which were more oncolytic than two or more reference strains across all cell types (Figure 5). If the precise cancer indication would not be known for an intended oHSV, these would be the strains to continue research with.

The reference strains had some differences in their oncolytic potentials in cell lines representing B-cell lymphoma and adenocarcinoma, but they were similar to each other in the neuroglioma cell line (Figure 5). In short, in the adenocarcinoma cell line, KOS was significantly less oncolytic than F and 17+, and in the lymphoma cells, both KOS and F were significantly less oncolytic than 17+. Therefore, in this set of cell types, 17+ was the most oncolytic of the three reference viruses. The 17+-derived neurovirulence gene mutant H1052 was different from 17+ in its replication in all studied cell lines, having a significantly lower cell-bound titer across the cell lines and a significantly lower titer for released virus in HCE cells (Figure 2), as well as being one of the most potent shedders across all three cell lines (Table 3). In addition to the major differences in replication, H1052 was different to 17+ in cytotoxicity in cells of lymphoma and of adenocarcinoma (Figure S1). H1052 was less oncolytic than 17+ in cells of adenocarcinoma, which may suggest that in adenocarcinomas the backbone or additional oncolytic transgenes are of very high relevance, as the necessary neurovirulence gene deletion decreases the oncolytic potential. Likewise, the choice of backbone would be of high interest in neuroglioma indications, as the oncolytic potential of H1052 was equal to that of its backbone (Figure S1). In the B-cell lymphoma cell line, H1052 was actually more oncolytic than 17+, despite the lower absolute replication (Figure 2), which could mean that there is high potential in oHSV treatment of hematological malignancies, as supported by recent literature [35,36].

Based on the replication profiles of the isolates in U373MG cells and HCE cells, potentially highly oncolytic strains could be at least V4, V5, and V12, as in both cell types, these strains had a phenotype with abundant replication (Table 4). V4 and V5 were indeed more cytotoxic than all reference strains in the B-cell lymphoma and adenocarcinoma cells, whereas V12 was more cytotoxic than F and KOS in the B-cell lymphoma cells and more cytotoxic than all reference strains in adenocarcinoma cells (Figure 5B). In addition to these isolates, the replication profile of V7 was of interest also in the neuroglioma cells, as it was identified to have highly efficient replication (Figure 2B). Still, all of these four potential isolates failed to demonstrate any statistical difference to 17+, F, or KOS in oncolytic potential in the neuroglioma cells (Figure 5A). Nevertheless, V7 was actually altogether the third most oncolytic strain in the cell line (Figure 5A).

In U373MG cells, the significantly more oncolytic strains were V13, V27, and V35, of which V27 was more oncolytic than all three reference strains (Figure 5A). V35, which was more oncolytic than 17+ and F, had a significantly higher cell-associated titer than 17+, but neither V27 nor V13 stood out by their viral shedding nor by cell-associated virus (Figure 2B, Table 3). Based on these results, for indications of treatment of neuroglioma with oHSV, V7, V13, V27, and V35 could prove as superior backbones in comparison to the reference viruses. Altogether, as H1052 and its backbone 17+ were similarly oncolytic in U373MG cells (Figure S1), the importance of the backbone in neuroglioma indications is highlighted. Furthermore, as the overall level of cytotoxicity was lower in neuroglioma cells than in the other cell types (Figure 5), further modifications, such as treatment related transgenes in the oHSV, may be highly valuable for oHSV efficacy in neuroglioma indications. In SW480 cells, all clinical isolates, except V17, were more oncolytic than all reference strains (Figure 5B), emphasizing the extreme potential of clinical isolates as backbones in the treatment of adenocarcinomas. Similarly, in Raji cells, six clinical isolates were more oncolytic than all reference strains (Figure 5C, Table 4). Therefore, also for B-cell lymphoma indications, there is an evident advantage to exploit clinical isolates as backbones for oHSVs.

## 5. Conclusions

In this study we characterized 36 clinical isolates from Finland with the aim of finding potential oncolytic backbones for oHSV development. The 36 isolates had varying characteristics for replication and spread (Figure 1 and Figure 2, Table 3), which appeared to depend on the cell type. Some viruses with a more extracellular or cell-bound phenotype across the cell types were identified, but they were rather rare. All studied isolates were sensitive to acyclovir (Figure 3), meaning that all isolates could be included in as future backbone based on their sensitivity to antiviral treatment. The isolates with more cell-associated phenotypes, such as V2, and rapid or strong replication, such as V4, V5, and V12, were also oncolytic (Figure 5). However, the most oncolytic isolates, V27 and V35, which were significantly more oncolytic than two or more of the reference strains in all cell lines (Figure 5), could not be identified via the replication characteristics (Figure 2). None of the found phenotypes were reflected in the genetic relatedness of the isolates, which was studied for two genes, US7 and UL27, encoding gI and gB (Figure 4). Rather than reflecting the phenotypes, the genotypes reflected the Asian and the European/North American distribution. Altogether, the clinical isolates were very oncolytic in contrast to reference viruses, especially in the adherent adenocarcinoma cell line (SW480) and in the lymphoma suspension cell line (Raji), emphasizing the advantages of clinical isolates even in very different types of cancer.

As a conclusion, clinical isolates appear to have obvious advantages over reference viruses as oHSV backbones. From a selection of 36 isolates, we were able to identify isolates with increased oncolytic potential in many types of cancer. All of the uncovered phenotypes were unrelated to the studied genotypes, and they were rather associated with the cell type. The future steps for this research are to perform full genome sequence of these clinical isolates and to continue research with selected oncolytic isolates by engineering their attenuated counterparts to confirm whether the observed increased oncolytic potential remains, as is suggested by previous literature.

## Figures and Tables

**Table 1:** Examples of oncolytic herpes simplex viruses (oHSVs) in recent or current clinical trials. As of 8 March 2022. For an expanded table with more comprehensive information, please see Supplementary Table S1.

Name	Parental Strain	Target ^1^	Phase	Status	Identifier	Ref.
**T-VEC** **(Imlygic)**	JS1 ^2^	Melanoma	II	Recruiting	NCT04330430	[5]
**M032**	F	Glioblastoma	I/II	Not yet recruiting	NCT05084430	[12]
**G207**	F	Pediatric brain tumors	II	Not yet recruiting	NCT04482933	[13]
**HSV1716**	17+	Mesothelioma	I/II	Completed	NCT01721018	[14]
**C-REV (HF10)**	HF	Melanoma	II	Completed	NCT03153085	[15]
**C134**	F	Glioblastoma	I	Recruiting	NCT03657576	[16]
**ONCR-177**	KOS	Melanoma	I	Recruiting	NCT04348916	[17]
**OrienX010**	CL1 ^2^	Melanoma	I	Recruiting	NCT04206358	[18]
**RP1**	RH018 ^2^	Melanoma	II	Recruiting	NCT03767348	[6]
**RP2**	RH018 ^2^	Cancer	I	Recruiting	NCT04336241	[6]
**RP3**	RH018 ^2^	Advanced Solid Tumor	I	Recruiting	NCT04735978	[6]
**rQNestin**	F	Brain cancer	I	Recruiting	NCT03152318	[19]
**VG161**	17+	Liver cancer	II	Not yet recruiting	NCT05223816	[20]
**NV1020**	F	Colorectal cancer	I/II	Completed	NCT00149396	[21]
**G47Δ**	F	Glioblastoma	II	Completed	UMIN000015995	[22]

^1^ Examples of clinical trials utilizing the oncolytic HSV. Many of the oncolytic HSVs are and have been in many clinical trials with various different oncologic indications. ^2^ Clinical isolate.

**Table 2:** Viruses used.

Virus	Description	Reference
V1-V36	Clinical isolates of herpes simplex virus type 1 (HSV-1)	This paper
HSV-1 Δ305	HSV-1 strain devoid of thymidine kinase	[26]
HSV-1 (17+)	HSV-1 reference strain	[28,29]
HSV-1 (KOS)	HSV-1 reference strain	[30]
HSV-1 (F)	HSV-1 reference strain	[31]
H1052	Derivative of HSV-1 17+ strain with pmCMV-eGFP and phCMV-LUC transgenes and neurovirulence gene (γ_1_34.5) deletions	[27]

**Table 3:** Proportions of extracellular to cell-associated virus. The mean proportions of extracellular to cell-associated virus were determined based on the data of Figure 2. Each column lists them for a cell line, and the highest 10 ratios within a cell line are colored yellow, whereas the lowest 10 are colored blue. Those highlighted in yellow represent viral strains with the highest extracellular shedding capability within a cell line, whereas those colored blue represent the most cell-associated viral strains. Each proportion is compared to that derived from 17+ from the same cell line. Any significant difference to 17+ is shown with a number that is in bold, in italics, and underlined.

	Vero	U373MG	HCE		Vero	U373MG	HCE
17+	12.0%	2.4%	7.6%	V17	6.4%	0.8%	3.3%
KOS	11.0%	0.2%	3.8%	V18	8.4%	0.1%	2.1%
F	25.8%	0.5%	2.4%	V19	***0.4%***	2.5%	5.4%
H1052	28.9%	1.7%	8.6%	V20	***3.3%***	0.9%	3.4%
V1	39.0%	3.0%	7.2%	V21	***4.9%***	0.7%	3.8%
V2	***2.9%***	0.2%	2.3%	V22	47.4%	1.0%	1.6%
V3	15.2%	1.5%	3.8%	V23	41.8%	1.4%	3.9%
V4	65.8%	3.3%	13.3%	V24	12.1%	2.6%	4.8%
V5	18.9%	1.3%	4.0%	V25	44.8%	1.1%	5.5%
V6	18.3%	6.0%	8.3%	V26	***5.6%***	1.2%	4.7%
V7	25.5%	*7.4%*	1.8%	V27	7.2%	1.2%	4.1%
V8	90.5%	1.8%	3.7%	V28	6.0%	1.4%	4.7%
V9	***4.7%***	2.4%	8.0%	V29	7.1%	0.1%	***1.3%***
V10	5.4%	0.2%	8.9%	V30	***3.3%***	0.2%	3.4%
V11	10.0%	6.0%	7.9%	V31	9.2%	1.7%	2.7%
V12	***5.5%***	***6.7%***	4.6%	V32	10.3%	0.2%	7.8%
V13	***0.6%***	2.2%	5.9%	V33	6.6%	0.1%	4.0%
V14	8.8%	1.8%	5.4%	V34	9.6%	0.6%	1.7%
V15	10.4%	0.7%	2.9%	V35	7.1%	2.7%	3.0%
V16	10.9%	0.3%	2.2%	V36	10.4%	3.5%	***1.5%***

**Table 4:** Summary of the significant differences found for each strain. An “X” marks a significant difference for each indicated phenotype in the indicated cell line.

	^1^ Higher Extracellular Virus Titer Than 17+			^1^ Higher Cell- Associated Titer Than 17+			^2^ Higher Proportional Shedding Than 17+			^2^ Higher Proportional Cell-Associated Virus Than 17+			^3^ More Oncolytic Than 17+			^3^ More Oncolytic Than F			^3^ More Oncolytic Than KOS		
	U373MG	HCE	Vero	U373MG	HCE	Vero	U373MG	HCE	Vero	U373MG	HCE	Vero	U373MG	Raji	SW480	U373MG	Raji	SW480	U373MG	Raji	SW480
**V1**				X											X		X	X		X	X
**V2**												X		X	X		X	X		X	X
**V3**	X														X		X	X		X	X
**V4**	X	X		X	X									X	X		X	X		X	X
**V5**	X	X		X	X									X	X		X	X		X	X
**V6**	X			X											X		X	X		X	X
**V7**	X			X	X		X								X		X	X		X	X
**V8**	X														X		X	X		X	X
**V9**												X			X			X			X
**V10**															X			X			X
**V11**	X														X			X			X
**V12**	X	X		X	X		X					X			X		X	X		X	X
**V13**		X										X	X		X			X			X
**V14**						X									X		X	X		X	X
**V15**															X		X	X		X	X
**V16**															X			X		X	X
**V17**																	X			X	X
**V18**															X			X			X
**V19**		X										X			X			X			X
**V20**												X			X		X	X		X	X
**V21**												X			X			X			X
**V22**			X												X			X			X
**V23**															X			X			X
**V24**					X										X		X	X		X	X
**V25**															X			X			X
**V26**				X								X			X			X			X
**V27**													X		X	X	X	X	X	X	X
**V28**															X		X	X		X	X
**V29**											X			X	X		X	X		X	X
**V30**												X			X			X			X
**V31**														X	X		X	X		X	X
**V32**															X		X	X		X	X
**V33**															X		X	X		X	X
**V34**															X		X	X		X	X
**V35**				X									X	X	X	X	X	X		X	X
**V36**				X	X						X				X		X	X		X	X

^1^ Data in Figure 2, ^2^ Data in Table 3, ^3^ Data in Figure 5.

## Data Availability

All relevant data are within the manuscript and its Supplementary Materials.

## Supplementary Materials

The following are available online at https://www.mdpi.com/article/10.3390/v14061290/s1, Figure S1: Cytotoxicity of reference strain 17+ compared to its neurovirulence-attenuated derivate H1052, Table S1: Oncolytic HSVs in recent or current clinical trials (expanded Table 1), File S1: UL27 alignment, File S2: US7 alignment.
