# Supplementary material for: The In Vitro Replication, Spread, and Oncolytic Potential of Finnish Circulating Strains of Herpes Simplex Virus Type 1
Source: Viruses. 2022 Jun 13;14(6):1290. doi: 10.3390/v14061290 (PMC9230972; doi:10.3390/v14061290)
Supplement: Supplementary file 1 [file viruses-14-01290-s001.zip › Supplementary Figure S1.pdf]

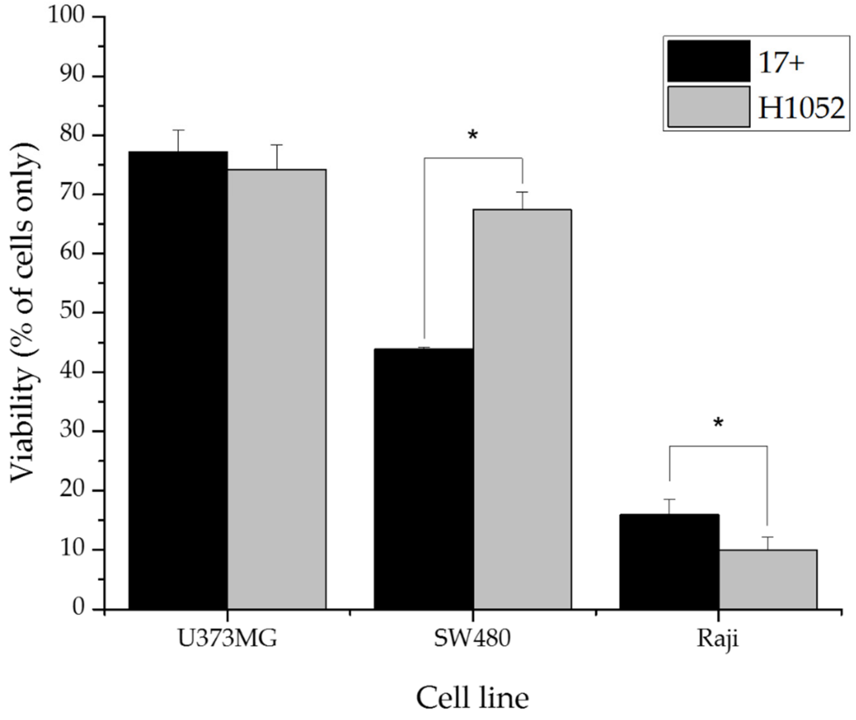


**Figure S1.** Cytotoxicity of the reference strain 17+ as compared to its neurovirulence-attenuated derivate H1052. The oncolytic effect of both strains was measured in three cancer cell lines at a 2 pfu/cell infection. The used cancerous cell lines, U373MG, SW480, and Raji, represented neuroglioma, colorectal adenocarcinoma, and B-cell lymphoma, respectively. The cellular viability was determined with Cell-Titer Glo at 96 hours post infection (hpi). The magnitude of the oncolytic effect was then determined by comparing results from the infected cells to the viability of uninfected control cells. The bars represent the relative viability of the cells, while the whiskers represent the standard deviation of the mean (N=4 per treatment).
