# Supplementary material for: The In Vitro Replication, Spread, and Oncolytic Potential of Finnish Circulating Strains of Herpes Simplex Virus Type 1
Source: Viruses. 2022 Jun 13;14(6):1290. doi: 10.3390/v14061290 (PMC9230972; doi:10.3390/v14061290)
Supplement: Supplementary file 1 [file viruses-14-01290-s001.zip › Supplementary Table S1.pdf]

Table S1. Examples of oncolytic HSVs in recent or current clinical trials. As of 8/3/2022. The table was compiled by a search in the ClinicalTrials.gov -database with the search term “HSV-1” and choosing the most recent clinical trial available for each vector.

oHSV					Example of a clinical study with the oHSV				
**Name**	**Transgene**	**Deletion**	**Parental strain**	**Reference**	**Target**	**Combinatorial**	**Phase**	**Status**	**Trial identifier**
T-VEC /Imlygic	GM-CSF	$\gamma_1 34.5$ (ICP34.5), U_S12 (ICP47)	JS1 (clinical isolate)	[1]	Melanoma (stage III and IV)	Checkpoint inhibitor (PD-1)	II	Recruiting	NCT04330430
M032	IL-12	$\gamma_1 34.5$ (ICP34.5)	F	[2]	Glioblastoma, astrocytoma, gliosarcoma	Checkpoint inhibitor (PD-1)	I/II	Not yet recruiting	NCT05084430
G207	LacZ	$\gamma_1 34.5$ (U_L39 (ICP6) disabled)	F	[3]	Pediatric brain tumors	Radiation	II	Not yet recruiting	NCT04482933
HSV1716	None	$\gamma_1 34.5$	17+	[4]	Mesothelioma	No	I/II	Completed	NCT01721018
HF10 /C-REV	None	UL56, LAT and $\gamma 34.5$ (ICP34.5) (Loss of expression of U_L43, $U_L49.5$, U_L55, U_L56)	HF	[5]	Melanoma	Checkpoint inhibitor (CTLA4)	II	Completed	NCT03153085
C134	Human cytomegalovirus (HCMV) gene IRS1	$\gamma_1 34.5$ (ICP34.5)	F	[6]	Brain cancer	No	I	Recruiting	NCT03657576
ONCR-177	IL12, FLT3LG, CCL4, and antagonists to PD-1 and CTLA-4	Tissue-specific miRNA mediated attenuation of U_L8, U_L54 (ICP27), ICP4, and the $\gamma_1 34.5$ (ICP34.5) genes	KOS	[7]	Melanoma	Checkpoint inhibitor (PD-1)	I	Recruiting	NCT04348916
OrienX010	GM-CSF	$\gamma_1 34.5$, U_S12 (ICP47), UL39 (ICP6)	CL1 (clinical isolate)	[8]	Melanoma	Checkpoint inhibitor (PD-1)	I	Recruiting	NCT04206358
RP1	GALV-GP R-, GM-CSF	$\gamma_1 34.5$ (ICP34.5), U_S12 (ICP47)	RH018 (clinical isolate)	[9]	Melanoma	Checkpoint inhibitor (PDL-1)	II	Recruiting	NCT03767348
RP2	GALV-GP R-, GM-CSF, anti-CTLA4			[9]	Cancer	Checkpoint inhibitor (PDL-1)	I	Recruiting	NCT04336241
RP3	GALV-GP R-, anti-			[9]	Advanced solid tumor	Checkpoint inhibitor (PD-1)	I	Recruiting	NCT04735978

	CTLA4, CD40L, 4-1BBL								
rQNestin	γ34.5 with a tumor-specific promoter	$\gamma_1$34.5 (ICP34.5), U_L39 (ICP6)	F	[10]	Brain cancer	Chemotherapy, biopsy	I	Recruiting	NCT03152318
VG161	IL-12, IL-15, IL-15RA and a PD-L1 blocker	$\gamma_1$34.5 (ICP35.4)	17+	[11]	Liver cancer	No	II	Not yet recruiting	NCT05223816
NV1020	Fragment of HSV-2 DNA (including gD, gG, gI, gJ, portion of gE), pα4-TK	One copy of $\gamma_1$34.5 (ICP34.5), α4 (ICP4), and α0 (ICP0), U_L56, (U_L24 disabled), U_L23 (TK)	F	[12]	Colorectal cancer	No	I/II	Completed	NCT00149396
G47Δ	LacZ	$\gamma_1$34.5 (ICP34.5), disabled U_L39 (ICP6), U_S12 (ICP47)	F	[13]	Glioblastoma	No	II	Completed	UMIN000015995

[1] B. L. Liu *et al.*, "ICP34.5 deleted herpes simplex virus with enhanced oncolytic, immune stimulating, and anti-tumour properties," *Gene Ther.*, vol. 10, no. 4, pp. 292–303, 2003, doi: 10.1038/SJ.GT.3301885.

[2] J. C. Roth *et al.*, "Evaluation of the Safety and Biodistribution of M032, an Attenuated Herpes Simplex Virus Type 1 Expressing hIL-12, After Intracerebral Administration to Aotus Nonhuman Primates," *Hum. Gene Ther. Clin. Dev.*, vol. 25, no. 1, p. 16, Mar. 2014, doi: 10.1089/HUMC.2013.201.

[3] T. Mineta, S. D. Rabkin, T. Yazaki, W. D. Hunter, and R. L. Martuza, "Attenuated multi-mutated herpes simplex virus-1 for the treatment of malignant gliomas," *Nat. Med.*, vol. 1, no. 9, pp. 938–943, 1995, doi: 10.1038/NM0995-938.

[4] A. MacLean, M. Ul-Fareed, L. Robertson, J. Harland, and S. Brown, "Herpes simplex virus type 1 deletion variants 1714 and 1716 pinpoint neurovirulence-related sequences in Glasgow strain 17+ between immediate early gene 1 and the 'a' sequence," *J. Gen. Virol.*, vol. 72 (Pt 3), no. 3, pp. 631–639, 1991, doi: 10.1099/0022-1317-72-3-631.

[5] I. R. Eissa *et al.*, "Genomic Signature of the Natural Oncolytic Herpes Simplex Virus HF10 and Its Therapeutic Role in Preclinical and Clinical Trials," *Front. Oncol.*, vol. 7, no. JUL, p. 149, Jul. 2017, doi: 10.3389/FONC.2017.00149.

[6] K. A. Cassady, "Human Cytomegalovirus TRS1 and IRS1 Gene Products Block the Double-Stranded-RNA-Activated Host Protein Shutoff Response Induced by Herpes Simplex Virus Type 1 Infection," *J. Virol.*, vol. 79, no. 14, p. 8707, Jul. 2005, doi: 10.1128/JVI.79.14.8707-8715.2005.

[7] B. B. Haines *et al.*, "ONCR-177, an Oncolytic HSV-1 Designed to Potently Activate Systemic Antitumor Immunity," *Cancer Immunol. Res.*, vol. 9, no. 3, pp. 291–308, Mar. 2021, doi: 10.1158/2326-6066.CIR-20-0609.

[8] J. Ren *et al.*, "Adaptive T cell responses induced by oncolytic Herpes Simplex Virus-granulocyte macrophage-colony-stimulating factor therapy expanded by dendritic cell and cytokine-induced killer cell adoptive therapy," *Oncoimmunology*, vol. 6, no. 4, Apr. 2016, doi: 10.1080/2162402X.2016.1264563.

[9] S. Thomas *et al.*, "Development of a new fusion-enhanced oncolytic immunotherapy platform based on herpes simplex virus type 1," *J. Immunother. Cancer*, vol. 7, no. 1, Aug. 2019, doi: 10.1186/S40425-019-0682-1.

[10] H. Kambara, H. Okano, E. A. Chiocca, and Y. Saeki, "An Oncolytic HSV-1 Mutant Expressing ICP34.5 under Control of a Nestin Promoter Increases Survival of Animals even when Symptomatic from a Brain Tumor," *Cancer Res.*, vol. 65, no. 7, pp. 2832–2839, Apr. 2005, doi: 10.1158/0008-5472.CAN-04-3227.

[11] D. V. Chouljenko *et al.*, "Induction of Durable Antitumor Response by a Novel Oncolytic Herpesvirus Expressing Multiple Immunomodulatory Transgenes," *Biomedicines*, vol. 8, no. 11, pp. 1–21, Nov. 2020, doi: 10.3390/BIOMEDICINES8110484.

[12] K. J. Kelly, J. Wong, and Y. Fong, "Herpes simplex virus NV1020 as a novel and promising therapy for hepatic malignancy," *Expert Opin. Investig. Drugs*, vol. 17, no. 7, p. 1105, Jul. 2008, doi: 10.1517/13543784.17.7.1105.

[13] T. Todo, R. L. Martuza, S. D. Rabkin, and P. A. Johnson, "Oncolytic herpes simplex virus vector with enhanced MHC class I presentation and tumor cell killing," *Proc. Natl. Acad. Sci. U. S. A.*, vol. 98, no. 11, pp. 6396–6401, May 2001, doi: 10.1073/PNAS.101136398/ASSET/4415FD06-4AC2-4099-97D2-B8A84F4AC423/ASSETS/GRAPHIC/PQ1011363006.JPEG.
